# Hearing Sensitivity, Cardiovascular Risk, and Neurocognitive Function: The Hispanic Community Health Study/Study of Latinos (HCHS/SOL)

**DOI:** 10.1001/jamaoto.2020.4835

**Published:** 2020-12-17

**Authors:** Ariana M. Stickel, Wassim Tarraf, Kathleen E. Bainbridge, Raymond P. Viviano, Martha Daviglus, Sumitrajit Dhar, Franklyn Gonzalez, Donglin Zeng, Hector M. González

**Affiliations:** 1 Department of Neurosciences and Shiley-Marcos Alzheimer’s Disease Research Center, University of California San Diego, La Jolla; 2 Institute of Gerontology, Wayne State University, Detroit, Michigan; 3 Department of Healthcare Sciences, Wayne State University, Detroit, Michigan; 4 National Institute on Deafness and Other Communication Disorders, Bethesda, Maryland; 5 Department of Psychology, Wayne State University, Detroit, Michigan; 6 Institute for Minority Health Research, University of Illinois at Chicago, College of Medicine, Chicago; 7 Department of Communication Sciences and Disorders, Northwestern University, Evanston, Illinois; 8 Department of Biostatistics, University of North Carolina, Chapel Hill, North Carolina

## Abstract

**Importance:**

Both cardiovascular disease risk and hearing impairment are associated with cognitive dysfunction. However, the combined influence of the 2 risk factors on cognition is not well characterized.

**Objective:**

To examine associations between hearing impairment, cardiovascular disease risk, and cognitive function.

**Design, Setting, and Participants:**

This population-based, prospective cohort, multisite cross-sectional analysis of baseline data collected between 2008 and 2011 as part of the Hispanic Community Health Study/Study of Latinos included 9623 Hispanic or Latino adults aged 45 to 74 years in New York, Chicago, Miami, and San Diego.

**Exposures:**

Hearing impairment of at least mild severity was defined as the pure tone average of 500, 1000, 2000, and 4000 Hz greater than 25 dB hearing level (dB HL) in the better ear. Our measure of cardiovascular disease risk was a latent class variable derived from body mass index, ankle-brachial index, low-density lipoprotein cholesterol, high-density lipoprotein cholesterol, triglycerides, fasting blood glucose, and the Framingham Cardiovascular Risk score.

**Main Outcomes and Measures:**

Results on Brief-Spanish English Verbal Learning Test (episodic learning and memory), and Word Fluency (verbal fluency), and Digit Symbol Subtest (processing speed/executive functioning), and a cognitive composite of the mentioned tests (overall cognition).

**Results:**

Participants (N = 9180) were 54.4% female and age 56.5 years on average. Hearing impairment was associated with poorer performance on all cognitive measures (global cognition: unstandardized β, −0.11; 95% CI, −0.16 to 0.07). Cardiovascular grouping (healthy, typical, high cardiovascular disease risk, and hyperglycemia) did not attenuate the associations between hearing impairment and cognition (global cognition: unstandardized β, −0.11; 95% CI, −0.15 to −0.06). However, cardiovascular grouping interacted with hearing impairment such that hyperglycemia in the context of hearing impairment exacerbated poor performance on learning and memory tasks (*F*_3_* *= 3.70 and *F*_3_* *= 2.92, respectively).

**Conclusions and Relevance:**

The findings of this cohort study suggest that hearing impairment increases the likelihood that individuals with excessively high glucose perform poorly on learning and memory tasks. Further research is needed to specify the mechanisms by which cardiovascular disease risk and hearing impairment are collectively associated with cognition.

## Key Points

**Question:**

Is hearing impairment associated with cardiovascular disease risk and cognitive function among Hispanic or Latino participants?

**Findings:**

In this cohort study of 9623 Hispanic/Latino adults, hearing impairment was associated with poorer cognitive performance on all tasks, and cardiovascular disease risk did not attenuate these relationships. Rather, hearing impairment modified the associations between cardiovascular disease risk and learning and memory; only among individuals with hearing impairment, being identified as having excessively high glucose was associated with poorer learning and memory relative to participants considered healthy individuals.

**Meaning:**

Hearing impairment may exacerbate the associations between high glucose and poorer cognition, particularly for learning and memory among Hispanic or Latino persons.

## Teaser

This cohort study examines associations between hearing impairment, cardiovascular disease risk, and cognitive function in the Hispanic/Latino population in 4 US cities.

## Introduction

Forty-seven percent of US adults aged 65 years and older report some degree of memory impairment,^1^ and an estimated 4.7 million people in this age group are living with Alzheimer disease.^2^ With the number of Alzheimer disease and related dementias (ADRD) cases expected to increase substantially by the year 2060, particularly among Hispanic or Latino patients,^3^ there is considerable interest in reducing the public health burden of modifiable risk factors for cognitive impairment in this group. Most attention has been placed on managing cardiovascular disease risk (CVDR) factors.^4^ The CVDR factors and mechanisms, including blood pressure dysregulation, arteriosclerosis, inflammation, and diabetes have been implicated across the spectrum of cognitive dysfunction from mild cognitive impairment to ADRD.^5,6^ Some data suggest that CVDR may have a stronger role in cognitive dysfunction and ADRD risk among Hispanic or Latino participants relative to non-Hispanic or Latino participants.^7,8,9^

More recently, hearing impairment has been associated with reduced cognitive function,^10,11,12,13,14^ incident cognitive impairment, and incident ADRD,^15^ but results are inconsistent.^13,16^ Although hearing impairment has a poor positive predictive value for cognitive impairment or dementia over a 10-year period,^12^ it has been suggested that treatment of hearing loss may reduce the risk of dementia.^17^ Despite high prevalence of both hearing impairment^18,19^ and ADRD^20^ among Hispanic or Latino participants, few studies have examined the connections between hearing impairment and cognition among this population.^10,21^ One exception is a recent study by Golub and colleagues^22^ which observed links between hearing impairment and cognition among a diverse group of middle-aged and older Hispanic or Latino participants.

Associations between hearing impairment and age-related cognitive decline may reflect a common cardiovascular pathway affecting nervous systems.^23^ As with the central nervous system, the auditory system is vulnerable to vascular pathophysiology.^24^ Epidemiologic evidence demonstrates that both hearing impairment and cognitive dysfunction are associated with CVDR factors (eg, hypertension, smoking).^19,25,26^ Several studies among older adults support an association between hearing loss and poorer cognition, independent of smoking, diabetes, and hypertension,^14,21,27^ but studies have not fully assessed vascular contributions. Importantly, Golub and colleagues^22^ did not find that controlling for cardiovascular disease attenuated the associations between hearing impairment and cognition among aging Hispanic or Latino participants. However, it is unclear as to whether having 2 risk factors for ADRD (ie, hearing impairment and cardiovascular disease) exacerbates poor cognitive performance.

The aim of this analysis was to expand on the study by Golub and colleagues^22^ of the associations between hearing impairment and cognition to include vascular contributions in the same cohort of diverse Hispanic or Latino participants. We hypothesized that (1) an inverse association between hearing impairment and cognitive function would be attenuated by controlling for CVDR and (2) the strength of the association between CVDR and cognitive function would vary by hearing impairment.

## Methods

### Study Participants

The Hispanic Community Health Study/Study of Latinos (HCHS/SOL) is a multisite (Bronx, Chicago, Miami, and San Diego), probability sampled, prospective cohort study of self-identified Hispanic or Latino participants aged 18 to 74 years. Study design, rationale, and implementation have been previously reported.^28,29^ In this study, we examined the oversampled middle-aged and older adults (45-74 years) who underwent neurocognitive testing from 2008 to 2011. Of these participants (n = 9623), we excluded 290 participants owing to missing data on model covariates (see sociodemographic covariates section). In addition, 153 participants did not have data to assess hearing impairment. Therefore, the primary analytical sample consisted of 9180 participants. Detailed presentation of missing data patterns and differences in sociodemographic and health characteristics are presented in eTables 1 and 2 in the Supplement. Institutional review boards at each participating site approved the study protocol. Participants provided informed consent.

### Cognitive Battery

Tests included the Brief-Spanish English Verbal Learning Test (B-SEVLT), Word Fluency (WF), and Digit Symbol Substitution (DSS) test.^30,31^ The B-SEVLT is an episodic verbal learning and memory test with 2 scores for: (1) learning (the summed total of correctly learned items across 3 trials [B-SEVLT-sum; range, 0-45]), and following an interference trial (2) memory (total correctly recalled items; range, 0-15). The WF is a phonemic verbal fluency test (total number of correctly generated words within 1 minute for the letters F and A). The DSS is a mental processing speed and executive functioning examination (range, 0-90 seconds). These HCHS/SOL cognitive tests, scoring procedures, and application to the HCHS/SOL data have been previously reported.^32,33^ All measures were z-score transformed ([score-mean]/standard deviation [SD]; using the tests’ probability weighted means and SDs) to facilitate score comparisons across tests using a common metric. A global cognitive score was derived by averaging the z-scores across the 4 domain-specific tests, as described herein.

### Hearing Impairment

Audiometric pure-tone air conduction hearing thresholds in decibels (hearing level) [dB HL] were obtained by trained, certified technicians for each ear at 500, 1000, 2000, 3000, 4000, 6000, and 8000 Hz using calibrated GSI-61 clinical audiometers with TDH-50 headphones in sound-attenuating booths. A modified Hughson-Westlake procedure was used in accordance with American Speech Language Association guidelines.^34^

For each individual and ear, we averaged air-conduction pure-tone thresholds measured at 500, 1000, 2000, and 4000 Hz to produce a pure-tone average (PTA), as a measure of speech-frequency hearing sensitivity. We identified participants with bilateral hearing impairment of at least mild severity using a greater than 25 dB HL PTA threshold in the better ear.^18^

### CVDR Indicators

**Figure 1.**
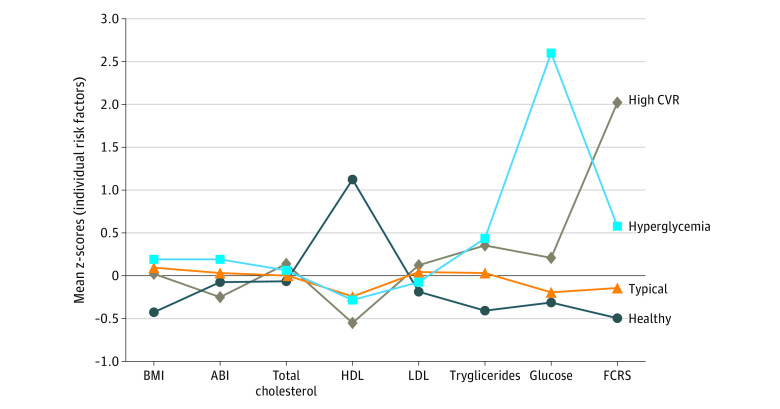
Risk Profiles of Latent Cardiovascular Risk Classes Derived From the Latent Profile Analyses ABI indicates ankle-brachial index; BMI, body mass index (calculated as weight in kilograms divided by height in meters squared); CVR, cardiovascular risk; FCRS, Framingham Cardiovascular Risk Score; HDL, high-density lipoproteins; LDL, low-density lipoproteins.

We modeled CVDR using latent profile analysis (LPA) techniques, which allow for identification of unobservable, data-derived subgroups. Latent profile analyses have been used extensively to model disease profiles (eg, cardiovascular), patient symptom experiences (eg, sleep), and patient-related outcomes (eg, heath services). The CVDR groups were estimated using 8 measures (Figure 1): body mass index (BMI, calculated as weight in kilograms divided by height in meters squared), ankle-brachial index (ABI), low-density lipoprotein (LDL), triglycerides (log transformed), and high-density lipoproteins (HDL), fasting blood glucose (FBG; log transformed), and Framingham Cardiovascular Risk Score (FCRS).^35,36^ Participant BMI was calculated as body weight in kilograms divided by height in meters squared.^37^ Participant ABI was based on the average ankle to arm systolic pressures as measured from the left and right sides. For each of the left and right sides, the ABI was calculated as the maximum systolic blood pressure in the posterior tibial artery or the dorsalis pedis artery in the same leg, divided by the maximum systolic blood pressures in the left and right brachial arteries. The overall composite ABI was then calculated for each participant as the minimum of the left- and right-side ABI.^38^ Fasting plasma lipid panels included total cholesterol (mg/dL), LDL, triglycerides, and HDL cholesterol, none of which required use of preparative ultracentrifuge. Participant LDL (mg/dL) was calculated using the Friedewald equation where LDL cholesterol = total cholesterol – HDL cholesterol – (triglycerides/5).^39,40^ Participant HDL (mg/dL) was measured with a direct magnesium/dextran sulfate method. Serum triglycerides (mg/dL) were measured via a Roche Modular P chemistry analyzer using a glycerol blanking enzymatic method.^41^ To capture glycemia, FBG (mg/dL) was measured using a hexokinase enzymatic method (Roche Diagnostics; https://www.cdc.gov/nchs/data/nhanes/nhanes_03_04/l10am_c_met_glucose.pdf). Finally, 10-year Framingham Risk Score was estimated using sex-specific published criteria.^42^

### Sociodemographic Covariates

We included the following list of confounding variables associated with cognitive function: age in years, sex (male, female), education (<12-year, 12-year, >12-year), Hispanic or Latino background (Dominicans, Cubans, Central Americans, Mexicans, Puerto Ricans, South Americans), annual household income measured in 4 brackets (≤$20 000, $20 001-$50 000,≥$50 001, no report), marital status (single, married/partnered, separated/divorced/widowed), Center for Epidemiologic Studies Depression Scale Revised (CES-D-10),^43^ and field center (Bronx, Chicago, San Diego, Miami).

### Statistical Analysis

Our analyses were conducted in 3 steps. First, we fit LPA models following standard procedures and sequentially assessed fitted class solutions (2 through 7; eTable 3 in the Supplement). A description of the LPA modeling steps, model fit assessment, and results are provided (eMethods 1 in the Supplement).

Second, we generated descriptive statistics to characterize the sample and examined the distributions of CVDR factors and diseases by hearing impairment status. Third, we used linear survey regression models to examine associations between hearing impairment and cognitive function and assess attenuations following adjustment for CVDR. For each outcome we tested (1) age, sex, education and center-adjusted, and (2) CVDR group-adjusted, and (3) full-covariates adjusted models and calculated and plotted average marginal estimates of cognitive performance by hearing impairment status using post hoc estimation techniques. Prior to assessing modifications in the associations between CVDR grouping and cognition by hearing impairment status, we fit survey logistic regression models to examine associations between CVDR groups and hearing impairment. Finally, we refit the models to test whether hearing impairment modified the association of CVDR classification and neurocognitive outcome by including an interaction term between hearing impairment and CVDR classification.

### Sensitivity

Three sensitivity analyses were conducted. Because the use of hearing aids may be a confounder in the relationship between hearing impairment and cognition, we conducted a sensitivity analysis repeating the described survey regression steps restricting the analytics sample to participants aged 45 to 74 years who denied hearing aid use. Second, we tested modifications in the associations between CVDR grouping and cognition by a continuous measurement of pure tone average in the better ear (measured at 0.5, 1, and 4 KHZ). Third, we also modeled a 3-class solution derived from the LPA to examine whether our findings were robust to a classification that included 3 groups only (rather than the adopted 4-class solution).

All analyses accounted for the complex design and probability weights of the HCHS/SOL.^44,45,46^ We used MPLUS statistical software (version 8.3, Muthen & Muthen) to generate the LPA models, Stata statistical software (version 16.1, StataCorp) to generate descriptive statistics and survey-adjusted regression models and visualization.

## Results

### CVDR Risk Groups

The CVDR profiles for each class membership (Figure 1) were consistent with (1) healthy (1883 [19.4%]) (2) typical (6189 [66.7%]) (3) high-CVDR (487 [7.5%]) and (4) hyperglycemia profiles (621 [6.5%]). The healthy group had mean BMI, triglycerides, glucose, and FCRS that were well below the population averages, and a mean HDL that was well above the population average. The typical group had CVDR profiles consistent with the population averages. The high-CVDR group had particularly more pronounced FCRS scores, and the hyperglycemia group had a glucose level that was excessively higher (mean [SD], 239.48 [80.24] mg/dL) (eTable 4 in the Supplement) than the population mean. The healthy group members were more likely to be women, have more than 12 years of education, and report a higher household income. Detailed descriptive statistics of the groups are provided in eTable 4 in the Supplement.

### Characteristics by Hearing Impairment

Individuals meeting criteria for hearing impairment were primarily male, had less than 12 years of education, and a yearly household income less than $20 000. Individuals with hearing impairment were older and had a worse cardiovascular profile on all the considered risk factors, with the exceptions of BMI and ABI (Table 1).

**Table 1.** Sociodemographic, Socioeconomic, and Health Characteristics of Target Population by Hearing Impairment Status

Characteristic	Hearing loss, % (SE)		
	No	Yes	Total
Sex			
Female	57.28 (0.75)	40.97 (2.00)	54.43 (0.70)
Education, y			
<12	37.02 (1.00)	52.84 (1.83)	39.79 (0.94)
12	21.92 (0.75)	17.83 (1.39)	21.21 (0.72)
>12	41.06 (1.02)	29.32 (1.89)	39.01 (0.93)
Center			
Bronx	25.43 (1.71)	24.72 (2.36)	25.31 (1.72)
Chicago	13.16 (0.89)	12.07 (1.33)	12.97 (0.89)
Miami	36.10 (2.42)	41.07 (3.23)	36.97 (2.46)
San Diego	25.32 (1.83)	22.14 (2.17)	24.76 (1.77)
Background			
Dominican	9.29 (0.72)	7.47 (1.16)	8.97 (0.72)
Central American	6.75 (0.47)	5.80 (0.71)	6.59 (0.43)
Cuban	26.49 (2.06)	33.01 (2.89)	27.63 (2.09)
Mexican	32.38 (1.81)	27.44 (2.33)	31.51 (1.77)
Puerto Rican	17.09 (1.05)	20.17 (1.67)	17.63 (1.02)
South American	5.68 (0.38)	4.41 (0.71)	5.45 (0.35)
Other	2.33 (0.36)	1.71 (0.41)	2.22 (0.30)
Income, $			
≤20 000	43.89 (1.13)	53.79 (2.10)	45.62 (1.12)
20 001-50 000	35.17 (0.88)	27.64 (1.66)	33.85 (0.82)
≥50 001	12.03 (0.94)	5.85 (0.94)	10.95 (0.83)
Not reported	8.92 (0.51)	12.72 (1.32)	9.58 (0.49)
Marital status			
Single	16.63 (0.65)	16.76 (1.48)	16.65 (0.63)
Married/partnered	53.92 (1.15)	50.96 (1.88)	53.41 (1.07)
Other	29.45 (0.92)	32.28 (1.79)	29.94 (0.85)
Hearing loss, mean (SD)			
Age, y	55.33 (9.51)	61.90 (8.94)	56.48 (9.90)
CESD-10	7.44 (7.86)	7.75 (7.35)	7.49 (7.78)
BMI	29.85 (6.90)	29.80 (5.96)	29.84 (6.73)
ABI	1.06 (0.16)	1.06 (0.18)	1.06 (0.16)
Total cholesterol	209.65 (54.15)	204.14 (51.86)	208.69 (53.89)
HDL	49.95 (16.62)	48.05 (14.78)	49.61 (16.33)
LDL	130.39 (46.76)	124.77 (43.80)	129.42 (46.37)
Triglycerides	148.70 (124.26)	164.86 (261.05)	151.52 (162.43)
Fasting blood glucose	109.10 (49.64)	116.48 (48.88)	110.39 (49.73)
FCRS	1.46 (1.54)	2.46 (1.90)	1.64 (1.69)

Abbreviations: ABI, ankle-brachial index; BMI, body mass index (calculated as weight in kilograms divided by height in meters squared); CESD, Center for Epidemiologic Studies Depression scale; FCRS, Framingham Cardiovascular Risk Score; HDL, high-density lipoproteins; LDL, low-density lipoproteins; SE, standard error.

### Association Between CVDR and Hearing Impairment

Individuals classified as high CVDR (OR, 1.48; 95% CI, 1.02-2.13) and hyperglycemia (OR, 1.64; 95% CI, 1.16-2.33) had 48% and 64% higher odds ratios of hearing impairment compared with those in the healthy group, after adjusting for age, sex, education, and field center. Typical and healthy groups were not statistically distinct (eTable 5 in the Supplement).

### Association Between Hearing Impairment, CVDR, and Cognitive Function

**Figure 2.**
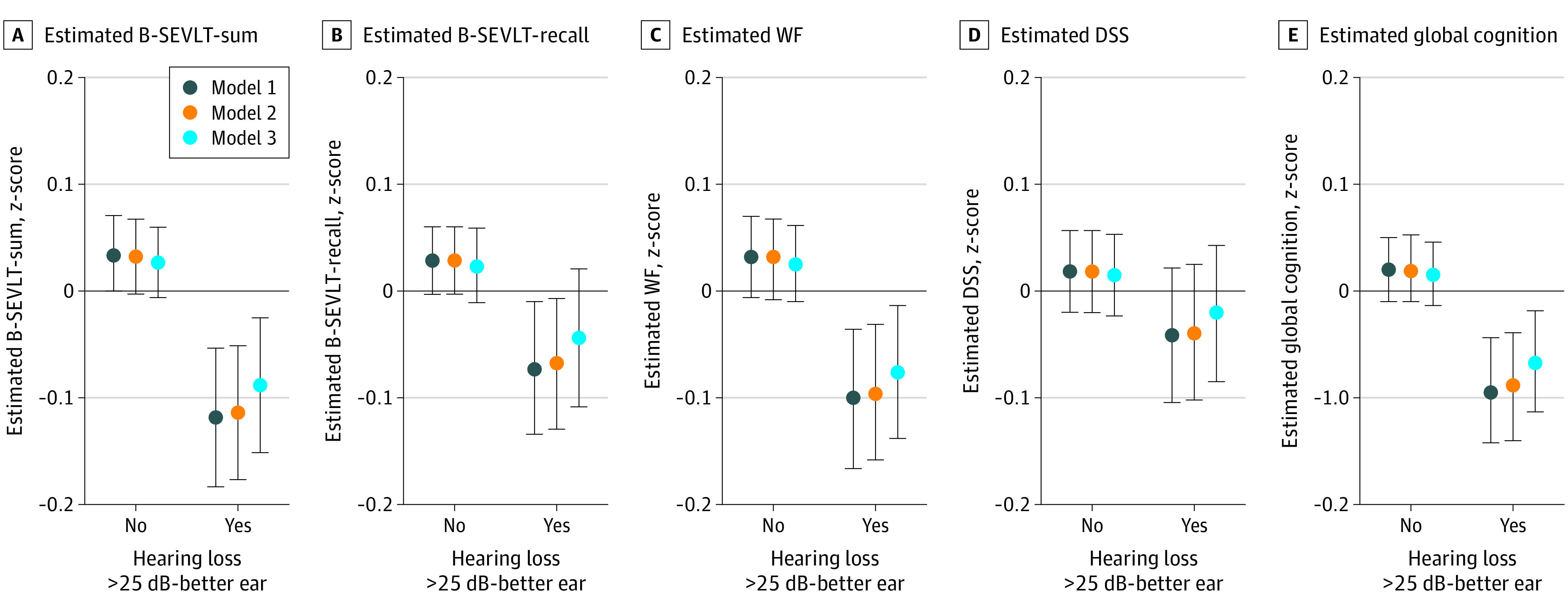
Cognitive Performance (z-Score) by Hearing Impairment Status^a^ B-SEVLT Indicates, Brief-Spanish English Verbal Learning Test; WF, word fluency; DSS, digit symbol substitution. ^a^Model 1 adjusted for age, sex, education, and Field Center. Model 2 includes additional adjustment for cardiovascular risk classification. Model 3 includes additional adjustment for Hispanic/Latino background, income, marital status, and depressive symptoms.

Hearing impairment (>25 dB) was associated with lower (z-score units) global cognition (B_GC_, –0.11 [SE, 0.02]) and consistently lower cognitive function on all considered cognitive domains (z-score units), including verbal learning and memory (B_B-SEVLT-Sum_, –0.15 [SE, 0.03]; B_B-SEVLT-Recall_, –0.10 [SE, 0.03]), verbal fluency (B_WF_, –0.13 [SE, 0.03]), and processing speed/executive functioning (B_DSS_, –0.06 [SE, 0.03]) (Figure 2). With the exception of the DSS, these associations were not attenuated through adjustment for the CVDR groups (Table 2).

**Table 2.** Associations Between Hearing Impairment, Cardiovascular Risk, and Cognitive Functioning^a^

Variable	B [95% CI]			
	M1	M2	M3	M4
B-SEVLT-sum				
High CVDR	–0.21 (–0.31 to –0.10)	NA	–0.19 (–0.29 to –0.08)	–0.16 (–0.26 to –0.06)
Hyperglycemia	–0.09 (–0.19 to –0.00)	NA	–0.08 (–0.17 to 0.01)	–0.04 (–0.13 to 0.04)
Healthy	[Reference]	NA	[Reference]	[Reference]
Typical	–0.05 (–0.11 to 0.01)	NA	–0.04 (–0.10 to 0.01)	–0.04 (–0.09 to 0.01)
≤25 dB (normal)	NA	[Reference]	[Reference]	[Reference]
>25 dB (mild or more)	NA	–0.15 (–0.22 to –0.09)	–0.15 (–0.21 to –0.08)	–0.12 (–0.18 to –0.05)
Intercept	1.08 (0.90 to 1.26)	1.04 (0.86 to 1.22)	1.00 (0.81 to 1.18)	1.08 (0.89 to 1.26)
B-SEVLT-recall				
High CVDR	–0.19 (–0.30 to –0.08)	NA	–0.18 (–0.29 to –0.07)	–0.16 (–0.26 to –0.05)
Hyperglycemia	–0.04 (–0.12 to 0.05)	NA	–0.03 (–0.11 to 0.06)	0.00 (–0.08 to 0.09)
Healthy	[Reference]	NA	[Reference]	[Reference]
Typical	–0.06 (–0.11 to –0.00)	NA	–0.06 (–0.11 to –0.00)	–0.05 (–0.11 to –0.00)
≤25 dB (normal)	NA	[Reference]	[Reference]	[Reference]
> 25 dB (mild or more)	NA	–0.10 (–0.16 to –0.04)	–0.10 (–0.16 to –0.03)	–0.07 (–0.13 to –0.00)
Intercept	1.02 (0.85 to 1.20)	1.01 (0.84 to 1.19)	0.98 (0.80 to 1.16)	0.99 (0.81 to 1.18)
Word fluency				
High CVDR	–0.25 (–0.37 to –0.13)	NA	–0.23 (–0.35 to –0.12)	–0.21 (–0.33 to –0.10)
Hyperglycemia	–0.16 (–0.27 to –0.05)	NA	–0.15 (–0.25 to –0.04)	–0.12 (–0.23 to –0.01)
Healthy	[Ref]	NA	[Reference]	[Reference]
Typical	–0.11 (–0.18 to –0.04)	NA	–0.11 (–0.18 to –0.04)	–0.10 (–0.17 to –0.03)
≤25 dB (normal)	NA	[Reference]	[Reference]	[Reference]
>25 dB (mild or more)	NA	–0.13 (–0.20 to –0.07)	–0.12 (–0.19 to –0.06)	–0.10 (–0.17 to –0.04)
Intercept	–0.15 (–0.37 to 0.08)	–0.22 (–0.43 to –0.01)	–0.22 (–0.45 to 0.00)	–0.34 (–0.55 to –0.12)
Digit symbol substitution				
High CVDR	–0.12 (–0.22 to –0.03)	NA	–0.12 (–0.21 to –0.02)	–0.08 (–0.17 to 0.00)
Hyperglycemia	–0.13 (–0.22 to –0.04)	NA	–0.12 (–0.21 to –0.03)	–0.09 (–0.17 to –0.00)
Healthy	[Reference]	NA	[Reference]	[Reference]
Typical	–0.08 (–0.13 to –0.02)	NA	–0.08 (–0.13 to –0.02)	–0.07 (–0.12 to –0.02)
≤25 dB (normal)	NA	[Reference]	[Reference]	[Reference]
>25 dB (mild or more)	NA	–0.06 (–0.12 to –0.00)	–0.06 (–0.11 to 0.00)	–0.04 (–0.09 to 0.02)
Intercept	1.32 (1.13 to 1.51)	1.25 (1.08 to 1.43)	1.28 (1.09 to 1.47)	1.05 (0.87 to 1.22)
Global cognition				
High CVDR	–0.19 (–0.27 to –0.12)	NA	–0.18 (–0.26 to –0.10)	–0.16 (–0.23 to –0.08)
Hyperglycemia	–0.10 (–0.17 to –0.03)	NA	–0.09 (–0.16 to –0.02)	–0.06 (–0.13 to 0.01)
Healthy	[Reference]	NA	[Reference]	[Reference]
Typical	–0.07 (–0.11 to –0.03)	NA	–0.07 (–0.11 to –0.03)	–0.07 (–0.10 to –0.03)
≤25 dB (normal)	NA	[Reference]	[Reference]	[Reference]
>25 dB (mild or more)	NA	–0.11 (–0.16 to –0.07)	–0.11 (–0.15 to –0.06)	–0.08 (–0.13 to –0.04)
Intercept	0.83 (0.69 to 0.97)	0.78 (0.65 to 0.91)	0.77 (0.63 to 0.91)	0.71 (0.58 to 0.84)

Abbreviations: B, unstandardized coefficient estimate; B-SEVLT, Brief–Spanish English Verbal Learning Test; CVDR, cardiovascular disease risk; dB, decibels; NA, not applicable.

^a^ All cognitive tests are normalized, and associations should be interpreted in z-score units. M1, M2, and M3 are age, sex, education, and Field Center adjusted, respectively. M4 includes additional adjustment for Hispanic/Latino background, income, marital status, and depressive symptoms.

In age, sex, education, and field-center adjusted models, the high-CVDR and hyperglycemia groups were associated with lower global cognitive function (B, –0.19 [SE, 0.04] and B, –0.09 [SE, 0.05], respectively). High CVDR classification was linked to consistently lower cognitive function on all considered domains; whereas hyperglycemia classification was associated with lower performance on verbal fluency and processing speed/executive functioning, only. These associations remained consistent after full covariate adjustment.

### Modifications in Association Between CVDR and Cognition by Hearing Impairment Status

**Figure 3.**
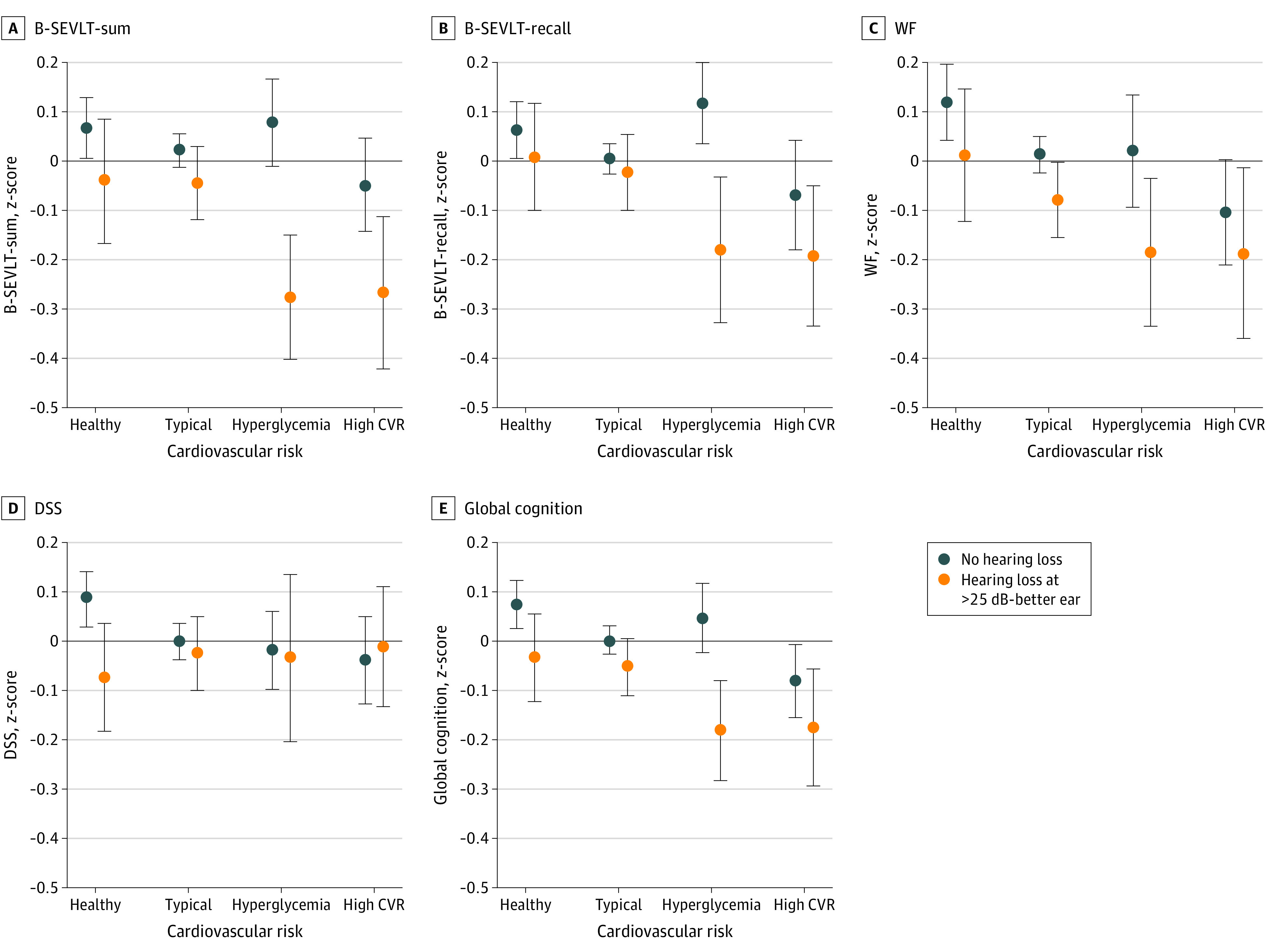
Modification in Cognitive Performance (z-Score) by Cardiovascular Risk Classification and Hearing Impairment Status^a^ CVR Indicates cardiovascular risk; B-SEVLT, Brief-Spanish English Verbal Learning Test; WF, word fluency; DSS, digit symbol substitution. ^a^Models include adjustment for age, sex, education, field center, Hispanic/Latino background, income, marital status, and depressive symptoms.

Adjusting for all covariates, we found evidence to support hearing impairment modifications that were specific to verbal learning (F, 3.70; df, 3) and memory (F, 2.92; df, 3) (eTable 6 in the Supplement). Modifications of CVDR grouping on cognition were notable only among individuals with hearing impairment. Hearing impairment status did not modify associations between CVDR grouping and global cognition, verbal fluency, or processing speed/executive functioning (Figure 3).

### Sensitivity Analyses

Results from the analyses focused on the target population of individuals with no history of hearing aid use were quantitatively equivalent to the main results. The results were consistent with our primary findings (eTables 7-9 in the Supplement). The results of the modification of the association of CVDR by continuous hearing were also consistent with the main findings (eTable 10 in the Supplement). In addition, we considered a competing LPA solution with 3 classes (high CVDR, hyperglycemia, and typical) (eFigure 1 in the Supplement). Results were also consistent with the estimates from the 4-class solution (eTables 11-14 and eFigures 2 and 3 in the Supplement). These findings provide an additional check on the robustness of our reported main results.

## Discussion

In this cohort of diverse middle-aged and older Hispanic or Latino participants, we found that after controlling for sociodemographic factors, excessive hyperglycemia was associated with poorer learning and memory only in the context of hearing impairment. In contrast, a high level of CVDR was associated with poorer cognition across several domains and regardless of hearing impairment status. Consistent with previous work,^22^ associations between hearing impairment and lower cognitive function were not fully explained by CVDR classification. Thus, we found limited support for CVDR factors as a common cause^47,48^ of hearing impairment and poor cognition. Despite this, CVDR was associated with increased risk for hearing impairment, suggesting potential direct and indirect pathways to minimize risk for cognitive impairment via treating CVDR. According to the 2020 report of the Lancet Commission, eliminating hearing loss alone could result in a nearly 8% reduction in ADRD cases.^15^ Perhaps more compelling, evidence suggests that hearing aid use is protective against poor cognitive functioning and cognitive decline among individuals with hearing impairment.^49,50^ Given that less than 5% of Hispanic or Latino participants with hearing impairment report hearing aid use,^51^ this suggests a considerable opportunity for intervention in the population expected to have the largest increase in ADRD cases in the US in the coming decades.^3^

Consistent with existing literature,^52,53,54^ we identified associations between hearing impairment and cognition. The present study sampled a relatively young (average age, 56 years) cohort, suggesting that mild hearing impairment (>25 dB) is associated with cognition prior to older adulthood. However, it is unclear if these early patterns are specific to Hispanic or Latino participants. Among a predominantly White sample,^52^ mild hearing impairment was only associated with faster rate of decline, not baseline cognitive performance, across a range of tasks. Others have detected poorer cognitive performance with moderate-to-severe, but not mild, hearing impairment.^53^ Alternatively, our sample was less educated than other samples,^52,53^ and lower education combined with mild hearing impairment has been associated with faster cognitive decline.^52^ Supporting this notion, mild hearing impairment was associated with lower cognition among Japanese individuals with lower education (>80% with ≤12 years of education).^54^ More recently, subclinical hearing impairment (≤15 dB) was associated with poorer cognition in both the HCHS/SOL and the somewhat higher educated National Health and Nutrition Examination Study cohorts,^21^ suggesting that this is not specific to Hispanic or Latino participants nor to those with low education.

Findings of this study extend the current literature on risk factors for cognitive impairment by showing the nuanced interplay between hearing impairment and CVDR. First, in line with previous HCHS/SOL findings,^18^ hyperglycemia classification was associated with increased hearing impairment. We also identified more robust associations between presence of multiple CVDR factors (ie, high CVDR) and hearing impairment relative to previous work. Our restricted age range (45-74 years vs 18-74 years in Cruickshanks et al^18^) may have increased the proportion of individuals with CVDR burden, which limited selective attrition due to earlier deaths, and thus increased our sensitivity to detect associations.

Second, although hearing impairment and CVDR were associated with one another, both factors appeared to have independent associations with cognition. Previous work has shown similar relationships between hearing impairment and cognition when controlling for CVDR, but did not examine the plausible role of CVDR.^21,22^ Given that CVDR did not attenuate the relationships between hearing impairment and cognition, our results do not support a cardiovascular common cause of hearing impairment and cognitive impairment.^47,48^ However, longitudinal data are required to confirm, and our results do not rule out the possibility of an altogether different factor that effects CVDR, hearing impairment, and cognition (eg, genetics).^55^

Third, we found that excessive hyperglycemia was associated with poorer learning and memory only in the presence of hearing impairment. This contrasts with our recent study demonstrating that diabetes was related to significant cognitive decline and mild cognitive impairment.^56^ However, the high-CVDR group was independently associated with poorer cognition across all domains, and such relationships were not contingent on or exacerbated by hearing impairment. Both hearing impairment^57,58^ and high CVDR^59^ have been associated with changes in brain structure, which may compromise cognitive functioning. Neuroimaging may aid in determining if the presence of multiple CVDRs are sufficient to affect brain structure and cognition, whereas hyperglycemia may be insufficient unless paired with another risk factor (eg, hearing impairment), thus constituting a “second hit”^60^ to the brain. Existing evidence suggests that the hippocampus and other medial temporal lobe structures that are integral to episodic learning and memory may be particularly susceptible to damage from diabetes^61^ as well as hearing impairment.^62^ Damage specific to the medial temporal lobe may explain why the combination of excessive hyperglycemia and hearing impairment was connected to poorer performance on learning and memory but not on other cognitive domains.

Importantly, the relationships between hearing and cognition were not cognitive domain specific, yet the modification by CVDR was specific to learning and memory. The latter is somewhat consistent with the information-degradation hypothesis,^63^ whereby tasks that require greater auditory load (ie, verbal learning and memory) may be more sensitive to hearing impairment. Among individuals classified as hyperglycemic and having hearing impairment, cognitive resources may be diverted away from cognition and toward more “effortful listening.”^53,64^ In addition, the only task that did not demonstrate a hearing impairment disadvantage was a nonverbal task of processing speed/executive functioning. Deal et al^53^ also were not able to detect hearing impairment-related differences in nonverbal (psychomotor and perceptual speed) tasks, whereas disadvantages were observed on baseline memory. Still, others^21,54^ including Golub and colleagues^22^ found that hearing impairment was associated with poorer performance on nonverbal tasks (ie, tasks that have low auditory load). Unlike Golub and colleagues,^22^ we did not find a robust association between hearing impairment and processing speed/executive functioning, possibly owing to differences in CVDR measures. All cardiometabolic factors incorporated into our analyses were measured in the clinical setting, and we used a data-driven approach to classifying CVDR. In addition, we extended the previous findings by controlling for key sociodemographic factors that have been shown to be related to hearing impairment and cognition in this population (eg, Hispanic/Latino background, socioeconomic status).^18,33,65,66^

A major strength of this study is extensive CVDR phenotyping among the most diverse cohort of Hispanic or Latino participants in the US. In addition, we replicated our findings using (1) a 3-class CVDR grouping and (2) only individuals who did not use hearing aids.

### Limitations

The conclusions drawn from our study are limited by several factors. First, our data are cross-sectional. Therefore, we were unable to test cognitive decline, and we are unable to make causal inferences. Second, although the use of audiometry to measure hearing impairment is a strength, we lack data on duration or history of hearing impairment. Third, we did not incorporate data on social isolation or depression as potential mediators between hearing impairment and cognitive decline.^67,68^ Fourth, although we controlled for several demographic factors, we did not examine interactions between hearing impairment and such factors. Finally, both audiometry and cognitive testing were completed in quiet (ideal hearing) environments, which enhance performance but may limit ecological validity.^69,70^

## Conclusions

In this study, hearing impairment was connected to poorer cognitive functioning among a representative sample of diverse middle-aged and older Hispanic or Latino participants. In addition, excessively high glucose was associated with poorer learning and memory only among individuals with hearing impairment. Our findings warrant longitudinal investigations into hearing impairment as a risk factor for cognitive decline and ADRD among diverse Hispanic or Latino patient populations.

## Notes

Supplement. **eMethods 1.** Latent profile analysis **eTable 1.** Missing data patterns **eTable 2.** Sociodemographic, socioeconomic, and health characteristics by missing data status **eTable 3.** Model fit statistics derived from sequential (2-7) latent profile analyses **eTable 4.** Sociodemographic, socioeconomic, and health characteristics of target population by four group cardiovascular risk classification **eTable 5.** Estimated odds ratios and 95% confidence intervals of the association between cardiovascular risk classification and hearing impairment **eTable 6.** Tests for significance of interaction between hearing impairment and cardiovascular risk classification **eTable 7.** Estimated odds ratios and 95% confidence intervals of the association between cardiovascular risk classification and hearing impairment, among individuals with no history of hearing aid use **eTable 8.** Associations between hearing impairment, cardiovascular risk classification and cognitive function, among individuals with no history of hearing aid use **eTable 9.** Tests for modifications in the effects of hearing impairment by cardiovascular risk classification, among individuals with no history of hearing aid use **eTable 10.** Tests for modifications in the associations between cognition and cardiovascular risk classification by continuous hearing **eTable 11.** Sociodemographic, socioeconomic, and health characteristics of target population by three group cardiovascular risk classification **eTable 12.** Estimated odds ratios and 95% confidence intervals of the association between three group cardiovascular risk classification and hearing impairment **eTable 13.** Associations between hearing impairment, three group cardiovascular risk classification and cognitive function **eTable 14.** Tests for significance of the modifications in the effects of hearing impairment by three group cardiovascular risk classification **eFigure 1.** Risk profiles of latent cardiovascular risk classes (3-class solution) derived from the latent profile analyses **eFigure 2.** Cognitive performance (z-score) by hearing impairment status **eFigure 3.** Modification in cognitive performance (z-score) by hearing impairment status and cardiovascular risk classification (3-class solution).
