# Supplementary material for: Hearing Sensitivity, Cardiovascular Risk, and Neurocognitive Function: The Hispanic Community Health Study/Study of Latinos (HCHS/SOL)
Source: JAMA Otolaryngol Head Neck Surg. 2020 Dec 17;147(4):377–87. doi: 10.1001/jamaoto.2020.4835 (PMC7747041; doi:10.1001/jamaoto.2020.4835)
Supplement: Supplement. — eMethods 1. Latent profile analysis eTable 1. Missing data patterns eTable 2. Sociodemographic, socioeconomic, and health characteristics by missing data status eTable 3. Model fit statistics derived from sequential (2-7) latent profile analyses eTable 4. Sociodemographic, socioeconomic, and health characteristics of target population by four group cardiovascular risk classification eTable 5. Estimated odds ratios and 95% confidence intervals of the association between cardiovascular risk classification and hearing impairment eTable 6. Tests for significance of interaction between hearing impairment and cardiovascular risk classification eTable 7. Estimated odds ratios and 95% confidence intervals of the association between cardiovascular risk classification and hearing impairment, among individuals with no history of hearing aid use eTable 8. Associations between hearing impairment, cardiovascular risk classification and cognitive function, among individuals with no history of hearing aid use eTable 9. Tests for modifications in the effects of hearing impairment by cardiovascular risk classification, among individuals with no history of hearing aid use eTable 10. Tests for modifications in the associations between cognition and cardiovascular risk classification by continuous hearing eTable 11. Sociodemographic, socioeconomic, and health characteristics of target population by three group cardiovascular risk classification eTable 12. Estimated odds ratios and 95% confidence intervals of the association between three group cardiovascular risk classification and hearing impairment eTable 13. Associations between hearing impairment, three group cardiovascular risk classification and cognitive function eTable 14. Tests for significance of the modifications in the effects of hearing impairment by three group cardiovascular risk classification eFigure 1. Risk profiles of latent cardiovascular risk classes (3-class solution) derived from the latent profile analyses eFigur [file jamaotolaryngolheadnecksurg-e204835-s001.pdf]

Supplemental Online Content

Stickel AM, Tarraf W, Bainbridge KE, et al. Hearing sensitivity, cardiovascular risk, and neurocognitive function: The Hispanic Community Health Study/Study of Latinos (HCHS/SOL). Published online December 17, 2020. *JAMA Otolaryngol Head Neck Surg*. doi:10.1001/jamaoto.2020.4835

eMethods 1. Latent profile analysis
eTable 1. Missing data patterns
eTable 2. Sociodemographic, socioeconomic, and health characteristics by missing data status
eTable 3. Model fit statistics derived from sequential (2-7) latent profile analyses
eTable 4. Sociodemographic, socioeconomic, and health characteristics of target population by four group cardiovascular risk classification
eTable 5. Estimated odds ratios and 95% confidence intervals of the association between cardiovascular risk classification and hearing impairment
eTable 6. Tests for significance of interaction between hearing impairment and cardiovascular risk classification
eTable 7. Estimated odds ratios and 95% confidence intervals of the association between cardiovascular risk classification and hearing impairment, among individuals with no history of hearing aid use
eTable 8. Associations between hearing impairment, cardiovascular risk classification and cognitive function, among individuals with no history of hearing aid use
eTable 9. Tests for modifications in the effects of hearing impairment by cardiovascular risk classification, among individuals with no history of hearing aid use
eTable 10. Tests for modifications in the associations between cognition and cardiovascular risk classification by continuous hearing
eTable 11. Sociodemographic, socioeconomic, and health characteristics of target population by three group cardiovascular risk classification
eTable 12. Estimated odds ratios and 95% confidence intervals of the association between three group cardiovascular risk classification and hearing impairment
eTable 13. Associations between hearing impairment, three group cardiovascular risk classification and cognitive function
eTable 14. Tests for significance of the modifications in the effects of hearing impairment by three group cardiovascular risk classification
eFigure 1. Risk profiles of latent cardiovascular risk classes (3-class solution) derived from the latent profile analyses
eFigure 2. Cognitive performance (z-score) by hearing impairment status
eFigure 3. Modification in cognitive performance (z-score) by hearing impairment status and cardiovascular risk classification (3-class solution).

© 2020 Stickel et al. *JAMA Otolaryngology–Head Neck Surgery*.

This supplemental material has been provided by the authors to give readers additional information about their work.

© 2020 Stickel et al. *JAMA Otolaryngology–Head Neck Surgery.*

eMethods 1. Latent profile analysis

Latent profile analysis (LPA) is a latent variable mixture modeling technique that identifies clusters, or latent subgroups, of participants in a dataset based on responses to a set of continuous variables. Theoretical and applied discussions of LPAs and their close equivalent for categorical variables are provided elsewhere.[1-3] Advantages of LPA over other clustering techniques, such as K-means clustering or hierarchical clustering, include the use of an explicit model to determine underlying subgroups, and the ability to account for uncertainty in these subgroups. As LPA is a probabilistic model, individuals receive a posterior probability for belonging to each subgroup. Furthermore, as LPA is a mixture modeling technique, the data is assumed to have been sampled from a population composed of a mix of probability distributions (multivariate normal). Classification steps in an analysis pipeline, such as LPA, allow for parsimonious representation of complex structures in a dataset. Individuals can be assigned to a derived subgroup based on their maximum posterior probability. Later analysis steps can then include these conceptually meaningful categories as predictor variables.

Here, we modeled cardiovascular disease risk profiles with LPA through estimates of body mass index (BMI), ankle-brachial Index (ABI), low-density lipoprotein (LDL), triglycerides (log transformed), and high-density lipoproteins (HDL), fasting blood glucose (FBG; log transformed), and Framingham Cardiovascular Risk Score (FCRS), see main text for description of data collection.

We conducted a series of LPAs using maximum likelihood estimation robust to non-normality and non-independence of observations in MPlus 8.3 accounting for the complex survey design structure of the data. We repeated the analysis specifying 2 up to 7 distinct latent profiles and then chose the model with the optimal number of profiles based on the Vuong-Lo-Mendell-Rubin Adjusted Likelihood Ratio Test (VLMR). We also evaluated model fit indices, class size, entropy, and conceptual meaningfulness of each profile to determine the optimal number of profiles to include in the final model. We determined that the four-profile solution provided optimal fit. The VLMR rejected the 5-profile solution over the 4-profile solution, $p = 0.44$, entropy of the 4-profile solution was greater than 0.8, and declines in the Akaike and Bayesian Information Criteria attenuated after the 4-profile solution. The 4-class solution was also easier to interpret compared to the others (eTable 3). Participants were assigned to one of these four derived latent CVDR profiles based on the highest membership posterior probability. We interpreted the profiles as Healthy (19.4%), Typical (66.7%), High CVDR (7.4), and Hyperglycemia (6.5%).

© 2020 Stickel et al. JAMA Otolaryngology–Head Neck Surgery.

eTable 1. Missing data patterns.[a]

			Missing (No, Yes)						
	**Percent Missing**	**Unweighted N**	**CVDR Class**	**Marital Status**	**Background**	**Education**	**FCRS**	**CESD-10**	**Hearing Loss**
**Patterns**	**(Complete) 95%**	9,180	No	No	No	No	No	No	No
	2%	153	No	No	No	No	No	No	Yes
	<1%	81	No	No	No	No	Yes	No	No
	<1%	78	No	No	No	No	No	Yes	Yes
	<1%	49	No	No	No	No	No	Yes	No
	<1%	14	No	No	No	Yes	No	No	No
	<1%	11	No	Yes	Yes	Yes	No	No	No
	<1%	11	No	Yes	No	No	No	No	No
	<1%	11	No	No	No	No	Yes	Yes	Yes
	<1%	10	No	No	Yes	No	No	No	No
	<1%	7	No	No	No	No	Yes	No	Yes
	<1%	6	No	No	No	No	Yes	Yes	No
	<1%	3	No	Yes	Yes	Yes	Yes	Yes	Yes
	<1%	2	Yes	No	No	No	Yes	Yes	Yes
	<1%	2	No	Yes	Yes	Yes	No	Yes	Yes
	<1%	2	No	No	Yes	Yes	No	No	No
	<1%	1	No	Yes	Yes	Yes	Yes	No	Yes
	<1%	1	No	Yes	Yes	Yes	No	Yes	No
	<1%	1	No	No	No	Yes	Yes	Yes	No

[a] Abbreviations: CVDR= cardiovascular disease risk; FCRS=Framingham Cardiovascular Risk Score; CESD = Center for Epidemiologic Studies Depression scale.

© 2020 Stickel et al. *JAMA Otolaryngology–Head Neck Surgery*.

eTable 2. Sociodemographic, socioeconomic, and health characteristics by missing data status. [a]

		Complete	Missing on Covariates	Missing on Covariates and Hearing	Total
**Sex**					
	Female	54.49 (0.68)	59.91 (5.12)	56.97 (6.64)	54.63 (0.68)
	Male	45.51 (0.68)	40.09 (5.12)	43.03 (6.64)	45.37 (0.68)
	Non-missing Unweighted N	9623			
**Education**					
	Less than HS	39.77 (0.93)	43.90 (5.56)	50.61 (7.11)	39.97 (0.91)
	HS or Equivalent	21.29 (0.71)	24.16 (4.52)	16.76 (4.77)	21.28 (0.70)
	More than HS	38.94 (0.92)	31.94 (5.52)	32.63 (6.51)	38.75 (0.90)
	Non-missing Unweighted N	9588			
**Field Center**					
	Bronx	25.93 (1.73)	39.50 (5.18)	73.96 (6.35)	26.78 (1.73)
	Chicago	12.78 (0.87)	12.62 (2.35)	8.00 (4.19)	12.72 (0.86)
	Miami	36.93 (2.45)	31.13 (5.37)	16.51 (5.40)	36.57 (2.41)
	San Diego	24.36 (1.74)	16.75 (3.86)	1.53 (0.85)	23.93 (1.70)
	Non-missing Unweighted N	9623			
**Hispanic/Latino Background**					
	Dominican	9.18 (0.74)	9.28 (2.83)	19.32 (5.09)	9.31 (0.74)
	Central	6.57 (0.42)	7.00 (2.26)	11.55 (6.56)	6.63 (0.42)
	Cuban	27.59 (2.08)	23.46 (5.79)	13.12 (5.20)	27.34 (2.06)
	Mexican	31.02 (1.75)	24.94 (4.40)	13.15 (4.90)	30.71 (1.71)
	Puerto-Rican	17.91 (1.03)	28.18 (5.01)	27.93 (5.28)	18.20 (1.02)
	South American	5.44 (0.35)	4.26 (2.01)	13.60 (5.01)	5.52 (0.34)
	Other	2.29 (0.30)	2.87 (1.84)	1.33 (0.96)	2.29 (0.29)
	Non-missing Unweighted N	9593			

[a] Abbreviations: SE = Standard error; SD = Standard deviation; CESD = Center for Epidemiologic Studies Depression scale.

© 2020 Stickel et al. *JAMA Otolaryngology–Head Neck Surgery.*

eTable 2. Sociodemographic, socioeconomic, and health characteristics by missing data status (continued).[a]					
		**Complete**	**Missing on Covariates**	**Missing on Covariates and Hearing**	**Total**
**Income**					
	<=$20000	45.85 (1.11)	48.53 (5.25)	55.82 (6.40)	46.02 (1.09)
	$20001-$50000	33.77 (0.82)	30.05 (5.14)	26.05 (5.59)	33.60 (0.81)
	>=$50001	10.87 (0.81)	6.64 (2.00)	4.13 (1.92)	10.71 (0.79)
	Not Reported	9.52 (0.49)	14.77 (4.26)	14.00 (4.10)	9.67 (0.48)
	Non-missing Unweighted N	9623			
**Marital Status**					
	Single	16.77 (0.62)	23.35 (4.96)	19.55 (6.56)	16.92 (0.62)
	Married/Partnered	53.17 (1.05)	45.83 (5.63)	48.78 (7.43)	52.99 (1.03)
	Separated/Divorced/Widowed	30.06 (0.84)	30.82 (4.84)	31.67 (6.36)	30.09 (0.82)
	Unweighted N	9594			
**Age**					
	Mean	56.45	57.29	57.62	56.48
	SD	9.89	10.73	11.13	9.93
	Non-missing Unweighted N	9623.00			
**CESD-10**					
	Mean	7.53	8.65	8.01	7.55
	SD	7.81	7.86	3.11	7.81
	Non-missing Unweighted N	9470			
**Cardiovascular Risk Class**					
	High CVR	7.41 (0.54)	5.85 (2.75)	8.47 (3.68)	7.40 (0.53)
	Hyperglycemia	6.45 (0.38)	2.35 (1.57)	3.97 (1.57)	6.34 (0.37)
	Healthy	19.40 (0.64)	15.17 (4.47)	22.21 (4.83)	19.36 (0.63)
	Typical	66.73 (0.72)	76.64 (5.03)	65.36 (6.55)	66.90 (0.71)

© 2020 Stickel et al. *JAMA Otolaryngology–Head Neck Surgery*.

	Non-missing Unweighted N	9621			

[a] Abbreviations: SE = Standard error; SD = Standard deviation; CESD = Center for Epidemiologic Studies Depression scale.

© 2020 Stickel et al. *JAMA Otolaryngology–Head Neck Surgery*.

eTable 3. Model fit statistics derived from sequential (2-7) latent profile analyses.[a]

Classes	Parameters	AIC	BIC	aBIC	AICC	Entropy	Lo-Mendell-Rubin Adjusted LRT
2	22	219882.894	220040.87	219970.957	219882.999	0.967	<0.0001
3	30	216563.434	216778.855	216683.52	216563.626	0.938	<0.0001
4	38	214257.711	214530.578	214409.82	214258.018	0.806	<0.0001
5	46	212433.15	212763.462	212617.281	212433.598	0.834	0.45
6	54	211465.612	211853.37	211681.766	211466.227	0.837	0.70
7	62	209836.83	210282.033	210085.007	209837.64	0.849	0.50

[a] Abbreviations: AIC = Akaike information criterion; BIC = Bayesian information Criterion; aBIC = Sample size adjusted BIC, AICC = Sample corrected AIC; LRT=Likelihood ratio test.

© 2020 Stickel et al. *JAMA Otolaryngology–Head Neck Surgery*.

eTable 4. Sociodemographic, socioeconomic, and health characteristics of target population by four group cardiovascular risk classification.[a]

		Cardiovascular Risk Groups				
		**High CVDR**	**Hyperglycemia**	**Healthy**	**Typical**	**Total**
		**%(SE)**	**%(SE)**	**%(SE)**	**%(SE)**	**%(SE)**
**Sex**						
	Female	21.16 (2.84)	49.05 (2.73)	73.31 (1.53)	53.19 (0.84)	54.43 (0.70)
**Education**						
	<12	46.77 (3.27)	47.50 (3.02)	34.66 (1.75)	39.74 (1.08)	39.79 (0.94)
	12	16.56 (2.00)	21.80 (2.39)	21.17 (1.33)	21.68 (0.94)	21.21 (0.72)
	>12	36.67 (3.02)	30.70 (2.78)	44.17 (1.77)	38.58 (1.08)	39.01 (0.93)
**Center**						
	Bronx	22.46 (3.31)	25.33 (2.66)	26.38 (2.09)	25.31 (1.85)	25.31 (1.72)
	Chicago	7.33 (1.40)	14.81 (1.84)	11.21 (0.95)	13.93 (1.02)	12.97 (0.89)
	Miami	53.57 (4.71)	30.81 (3.58)	35.04 (2.79)	36.26 (2.41)	36.97 (2.46)
	San Diego	16.63 (3.10)	29.06 (3.56)	27.36 (2.14)	24.50 (1.87)	24.76 (1.77)
**Background**						
	Dominican	6.35 (1.46)	7.66 (1.49)	10.17 (0.99)	9.04 (0.79)	8.97 (0.72)
	Central	6.05 (1.33)	7.81 (1.29)	5.96 (0.61)	6.71 (0.50)	6.59 (0.43)
	Cuban	44.12 (4.32)	21.72 (3.24)	24.64 (2.32)	27.22 (2.06)	27.63 (2.09)
	Mexican	19.16 (3.20)	38.65 (3.51)	32.29 (2.09)	31.98 (1.85)	31.51 (1.77)
	Puerto-Rican	18.04 (2.51)	19.65 (2.23)	18.31 (1.87)	17.19 (1.05)	17.63 (1.02)
	South American	3.23 (0.77)	3.51 (0.89)	6.42 (0.80)	5.61 (0.40)	5.45 (0.35)
	Other	3.05 (0.95)	1.00 (0.52)	2.21 (0.50)	2.25 (0.42)	2.22 (0.30)

[a] Abbreviations: SE = Standard error; SD = Standard deviation; CESD = Center for Epidemiologic Studies Depression scale; BMI = Body mass index; ABI = Ankle-brachial index; HDL = High-density lipoproteins; LDL = Low-density lipoproteins; FCRS = Framingham Cardiovascular Risk Score

© 2020 Stickel et al. *JAMA Otolaryngology–Head Neck Surgery*.

eTable 4. Sociodemographic, socioeconomic, and health characteristics of target population by four group cardiovascular risk classification (continued).[a]

		Cardiovascular Risk Groups				
		**High CVDR**	**Hyperglycemia**	**Healthy**	**Typical**	**Total**
		**%(SE)**	**%(SE)**	**%(SE)**	**%(SE)**	**%(SE)**
**Income**						
	<=$20000	55.35 (3.16)	53.10 (2.97)	44.19 (1.98)	44.22 (1.18)	45.62 (1.12)
	$20001-$50000	27.63 (2.59)	30.79 (2.77)	32.31 (1.66)	35.29 (1.02)	33.85 (0.82)
	>=$50001	6.09 (1.59)	7.05 (1.39)	13.12 (1.43)	11.25 (0.94)	10.95 (0.83)
	Not Reported	10.93 (1.97)	9.06 (1.41)	10.38 (1.04)	9.25 (0.62)	9.58 (0.49)
**Marital Status**						
	Single	15.78 (2.33)	17.45 (1.94)	17.54 (1.19)	16.42 (0.72)	16.65 (0.63)
	Married/Partnered	52.17 (3.16)	54.04 (2.74)	47.66 (1.94)	55.15 (1.27)	53.41 (1.07)
	Other	32.06 (2.87)	28.51 (2.48)	34.79 (1.74)	28.43 (1.02)	29.94 (0.85)
		**Mean (SD)**	**Mean (SD)**	**Mean (SD)**	**Mean (SD)**	**Mean (SD)**
**Age**		65.21 (6.17)	56.35 (9.70)	55.24 (9.79)	55.87 (9.64)	56.48 (9.90)
**CESD-10**		7.22 (6.07)	8.49 (8.27)	7.34 (8.13)	7.47 (7.81)	7.49 (7.78)
**BMI**		29.99 (4.93)	31.18 (7.65)	27.00 (6.15)	30.53 (6.66)	29.84 (6.73)
**ABI**		1.02 (0.18)	1.09 (0.23)	1.05 (0.15)	1.06 (0.15)	1.06 (0.16)
**HDL**		40.53 (10.28)	45.04 (14.24)	68.02 (15.17)	45.72 (10.83)	49.61 (16.33)
**LDL**		134.83 (40.85)	125.56 (55.35)	120.37 (41.77)	131.87 (46.60)	129.42 (46.37)
**Triglycerides**		211.15 (170.38)	224.96 (466.43)	81.84 (35.47)	157.98 (105.30)	151.52 (162.43)
**Fasting Blood Glucose**		120.65 (27.23)	239.48 (80.24)	94.93 (14.02)	101.25 (17.63)	110.39 (49.73)
**FCRS**		5.05 (1.30)	2.61 (1.76)	0.80 (0.77)	1.41 (1.05)	1.64 (1.69)

[a] Abbreviations: SE = Standard error; SD = Standard deviation; CESD = Center for Epidemiologic Studies Depression scale; BMI = Body mass index; ABI = Ankle-brachial index; HDL = High-density lipoproteins; LDL = Low-density lipoproteins; FCRS = Framingham Cardiovascular Risk Score

© 2020 Stickel et al. *JAMA Otolaryngology–Head Neck Surgery*.

eTable 5. Estimated odds ratios and 95% confidence intervals of the association between cardiovascular risk classification and hearing impairment.[a]

		Hearing Loss >25 dB
		**OR [95%CI]**
**Cardiovascular Risk Groups**		
	High CVDR	1.48 [1.02,2.13]
	Hyperglycemia	1.64 [1.16,2.33]
	Healthy	ref
	Typical	1.18 [0.95,1.48]
**Age (in years)**		1.10 [1.09,1.12]
**Sex**		
	Male	2.11 [1.73,2.59]
**Education (in years)**		
	<12	ref
	12	0.73 [0.58,0.91]
	>12	0.59 [0.48,0.73]
**Center**		
	Bronx	ref
	Chicago	0.95 [0.74,1.21]
	Miami	1.04 [0.83,1.30]
	San Diego	0.98 [0.76,1.27]
**Intercept**		0.00 [0.00, 0.00]

[a] Abbreviations: OR = Odds ratio; CI = Confidence interval; CVDR = Cardiovascular risk; dB = Decibels

© 2020 Stickel et al. *JAMA Otolaryngology–Head Neck Surgery.*

eTable 6. Tests for significance of interaction between hearing impairment and cardiovascular risk classification.[a]

Cognitive Test	F	df
B-SEVLT-Sum	3.697	3
B-SEVLT-Recall	2.918	3
Word Fluency	0.420	3
Digit Symbol Substitution	1.867	3
Global Cognition	2.137	3

[a] Abbreviations: B-SEVLT = Brief–Spanish English Verbal Learning Test; df = Degree of freedom

© 2020 Stickel et al. *JAMA Otolaryngology–Head Neck Surgery.*

eTable 7. Estimated odds ratios and 95% confidence intervals of the association between cardiovascular risk classification and hearing impairment, among individuals with no history of hearing aid use.[a]

		**Hearing Loss >25 dB**
		**OR [95%CI]**
**Cardiovascular Risk Groups**		
	High CVDR	1.55 [1.06,2.28]
	Hyperglycemia	1.72 [1.20,2.47]
	Healthy	ref
	Typical	1.19 [0.94,1.50]
**Age**		1.10 [1.09,1.11]
**Sex**		
	Male	2.13 [1.73,2.63]
**Education**		
	<12	ref
	12	0.70 [0.56,0.89]
	>12	0.57 [0.45,0.71]
**Center**		
	Bronx	ref
	Chicago	0.96 [0.74,1.23]
	Miami	1.08 [0.85,1.36]
	San Diego	1.00 [0.76,1.30]

[a] Abbreviations: OR = Odds ratio; CI = Confidence Interval; CVDR = Cardiovascular risk; dB = decibels

© 2020 Stickel et al. *JAMA Otolaryngology–Head Neck Surgery*.

eTable 8. Associations between hearing impairment, cardiovascular risk classification and cognitive function, among individuals with no history of hearing aid use.[a, b]

		M1	M2	M3	M4
		B [95% CI]	B [95% CI]	B [95% CI]	B [95% CI]
**B-SEVLT-Sum**					
	High CVDR	-0.20 [-0.31;-0.09]	n/a	-0.18 [-0.29;-0.07]	-0.15 [-0.25;-0.05]
	Hyperglycemia	-0.09 [-0.19;-0.00]	n/a	-0.08 [-0.17;0.01]	-0.04 [-0.13;0.04]
	Healthy	0.00 [0.00;0.00]	n/a	ref	ref
	Typical	-0.05 [-0.10;0.01]	n/a	-0.04 [-0.10;0.02]	-0.04 [-0.09;0.02]
	≤25 dB (Normal)	n/a	ref	ref	ref
	>25 dB (Mild or more)	n/a	-0.15 [-0.22;-0.08]	-0.14 [-0.21;-0.07]	-0.11 [-0.18;-0.05]
	Intercept	1.09 [0.91;1.27]	1.06 [0.88;1.23]	1.01 [0.83;1.20]	1.09 [0.91;1.27]
**B-SEVLT-Recall**					
	High CVDR	-0.18 [-0.29;-0.07]	n/a	-0.17 [-0.28;-0.06]	-0.14 [-0.25;-0.04]
	Hyperglycemia	-0.03 [-0.12;0.05]	n/a	-0.02 [-0.11;0.06]	0.00 [-0.08;0.09]
	Healthy	0.00 [0.00;0.00]	n/a	ref	ref
	Typical	-0.06 [-0.11;-0.00]	n/a	-0.06 [-0.11;-0.00]	-0.06 [-0.11;-0.00]
	≤25 dB (Normal)	n/a	ref	ref	ref
	>25 dB (Mild or more)	n/a	-0.10 [-0.16;-0.04]	-0.10 [-0.16;-0.03]	-0.07 [-0.13;-0.00]
	Intercept	1.03 [0.85;1.20]	1.02 [0.84;1.19]	0.99 [0.81;1.16]	1.00 [0.81;1.18]
**Word Fluency**					
	High CVDR	-0.25 [-0.37;-0.13]	n/a	-0.24 [-0.36;-0.11]	-0.21 [-0.34;-0.09]
	Hyperglycemia	-0.16 [-0.27;-0.05]	n/a	-0.15 [-0.26;-0.04]	-0.12 [-0.23;-0.01]
	Healthy	0.00 [0.00;0.00]	n/a	ref	ref
	Typical	-0.11 [-0.18;-0.04]	n/a	-0.11 [-0.18;-0.03]	-0.10 [-0.17;-0.03]
	≤25 dB (Normal)	n/a	ref	ref	ref
	>25 dB (Mild or more)	n/a	-0.13 [-0.20;-0.07]	-0.13 [-0.19;-0.06]	-0.10 [-0.17;-0.04]
	Intercept	-0.16 [-0.39;0.06]	-0.24 [-0.45;-0.03]	-0.24 [-0.46;-0.01]	-0.34 [-0.56;-0.12]

© 2020 Stickel et al. *JAMA Otolaryngology–Head Neck Surgery.*

**Digit Symbol Substitution**					
	High CVDR	-0.11 [-0.21;-0.02]	n/a	-0.11 [-0.21;-0.01]	-0.08 [-0.17;0.02]
	Hyperglycemia	-0.12 [-0.21;-0.03]	n/a	-0.12 [-0.21;-0.03]	-0.08 [-0.17;0.00]
	Healthy	0.00 [0.00;0.00]	n/a	0.00 [0.00;0.00]	0.00 [0.00;0.00]
	Typical	-0.08 [-0.13;-0.02]	n/a	-0.08 [-0.13;-0.02]	-0.07 [-0.12;-0.02]
	≤25 dB (Normal)	n/a	0.00 [0.00;0.00]	0.00 [0.00;0.00]	0.00 [0.00;0.00]
	>25 dB (Mild or more)	n/a	-0.06 [-0.12;-0.00]	-0.06 [-0.12;0.00]	-0.04 [-0.09;0.02]
	Intercept	1.34 [1.14;1.53]	1.27 [1.09;1.45]	1.30 [1.10;1.49]	1.06 [0.89;1.24]
**Global Cognition**					
	High CVDR	-0.19 [-0.27;-0.11]	n/a	-0.18 [-0.26;-0.10]	-0.15 [-0.22;-0.07]
	Hyperglycemia	-0.10 [-0.17;-0.03]	n/a	-0.09 [-0.16;-0.02]	-0.06 [-0.13;0.01]
	Healthy	0.00 [0.00;0.00]	n/a	ref	ref
	Typical	-0.07 [-0.11;-0.03]	n/a	-0.07 [-0.11;-0.03]	-0.06 [-0.10;-0.03]
	≤25 dB (Normal)	n/a	ref	ref	ref
	>25 dB (Mild or more)	n/a	-0.11 [-0.16;-0.07]	-0.11 [-0.15;-0.06]	-0.08 [-0.13;-0.04]
	Intercept	0.83 [0.69;0.97]	0.79 [0.65;0.92]	0.77 [0.63;0.91]	0.71 [0.59;0.84]

[a] Abbreviations: B = Unstandardized coefficient estimate; CI = Confidence interval; ref = Reference category; n/a = not applicable; B-SEVLT = Brief–Spanish English Verbal Learning Test; dB = decibels; CVDR = Cardiovascular disease risk

[b] All cognitive tests are normalized, and associations should be interpreted in z-score units. M1, M2, and M3 are age, sex, education, and Field Center adjusted. M4 includes additional adjustment for Hispanic/Latino background, income, marital status, and depressive symptoms.

© 2020 Stickel et al. *JAMA Otolaryngology–Head Neck Surgery*.

eTable 9. Tests for modifications in the effects of hearing impairment by cardiovascular risk classification, among individuals with no history of hearing aid use.[a]

**Cognitive Test**	**F**	**df**
B-SEVLT-Sum	4.086	3
B-SEVLT-Recall	2.941	3
Word Fluency	0.500	3
Digit Symbol Substitution	1.844	3
Global Cognition	2.204	3

[a] Abbreviations: B-SEVLT = Brief–Spanish English Verbal Learning Test; df = Degree of freedom

© 2020 Stickel et al. *JAMA Otolaryngology–Head Neck Surgery.*

eTable 10. Tests for modifications in the associations between cognition and cardiovascular risk classification by continuous hearing.[a]

Cognitive Test	F	df
B-SEVLT-Sum	2.681	3
B-SEVLT-Recall	3.481	3
Word Fluency	0.152	3
Digit Symbol Substitution	0.503	3
Global Cognition	1.709	3

[a] Abbreviations: B-SEVLT = Brief–Spanish English Verbal Learning Test; df = Degree of freedom

© 2020 Stickel et al. *JAMA Otolaryngology–Head Neck Surgery*.

Table 11. Sociodemographic, socioeconomic, and health characteristics of target population by three group cardiovascular risk classification.[a]

		Cardiovascular Risk Groups			
		**Typical**	**High CVDR**	**Hyperglycemia**	**Total**
		**%(SE)**	**%(SE)**	**%(SE)**	**%(SE)**
**Sex**					
	Female	57.94 (0.73)	22.76 (2.69)	49.64 (2.77)	54.43 (0.70)
**Education**					
	<12	38.50 (0.97)	46.26 (3.19)	48.13 (3.04)	39.79 (0.94)
	12	21.61 (0.81)	16.84 (1.94)	21.63 (2.38)	21.21 (0.72)
	>12	39.89 (0.98)	36.91 (2.96)	30.25 (2.76)	39.01 (0.93)
**Center**					
	Bronx	25.56 (1.72)	22.65 (3.08)	25.42 (2.67)	25.31 (1.72)
	Chicago	13.40 (0.93)	7.41 (1.27)	14.52 (1.82)	12.97 (0.89)
	Miami	35.83 (2.34)	53.56 (4.43)	30.27 (3.60)	36.97 (2.46)
	San Diego	25.21 (1.79)	16.38 (2.80)	29.79 (3.56)	24.76 (1.77)
**Background**					
	Dominican	9.31 (0.74)	6.35 (1.36)	7.90 (1.52)	8.97 (0.72)
	Central	6.56 (0.44)	5.96 (1.24)	7.80 (1.29)	6.59 (0.43)
	Cuban	26.45 (1.97)	44.50 (4.11)	21.15 (3.27)	27.63 (2.09)
	Mexican	32.17 (1.77)	18.99 (2.91)	39.18 (3.52)	31.51 (1.77)
	Puerto-Rican	17.43 (1.03)	18.23 (2.33)	19.49 (2.22)	17.63 (1.02)
	South American	5.85 (0.39)	2.98 (0.69)	3.48 (0.88)	5.45 (0.35)
	Other	2.24 (0.35)	3.00 (0.85)	0.99 (0.52)	2.22 (0.30)

[a] Abbreviations: SE = Standard error; SD = Standard deviation; CESD = Center for Epidemiologic Studies Depression scale; BMI = Body mass index; ABI = Ankle-brachial index; HDL = High-density lipoproteins; LDL = Low-density lipoproteins; FCRS = Framingham Cardiovascular Risk Score

© 2020 Stickel et al. *JAMA Otolaryngology–Head Neck Surgery*.

eTable 11. Sociodemographic, socioeconomic, and health characteristics of target population by three group cardiovascular risk classification (continued).[a]

		Cardiovascular Risk Groups			
		**Typical**	**High CVDR**	**Hyperglycemia**	**Total**
		**%(SE)**	**%(SE)**	**%(SE)**	**%(SE)**
**Income**					
	<=$20000	44.04 (1.16)	55.64 (2.89)	53.17 (2.96)	45.62 (1.12)
	$20001-$50000	34.78 (0.92)	26.82 (2.34)	30.89 (2.74)	33.85 (0.82)
	>=$50001	11.78 (0.90)	5.70 (1.42)	6.95 (1.38)	10.95 (0.83)
	Not Reported	9.40 (0.56)	11.84 (1.91)	8.99 (1.40)	9.58 (0.49)
**Marital Status**					
	Single	16.73 (0.65)	15.32 (2.13)	17.41 (1.94)	16.65 (0.63)
	Married/Partnered	53.42 (1.18)	52.88 (2.92)	53.91 (2.76)	53.41 (1.07)
	Other	29.85 (0.95)	31.80 (2.67)	28.68 (2.47)	29.94 (0.85)
		**Mean (SD)**	**Mean (SD)**	**Mean (SD)**	**Mean (SD)**
**Age**		55.66 (9.67)	64.93 (6.32)	56.26 (9.61)	56.48 (9.90)
**CESD-10**		7.44 (7.89)	7.30 (6.26)	8.43 (8.17)	7.49 (7.78)
**BMI**		29.72 (6.81)	30.18 (5.19)	31.11 (7.54)	29.84 (6.73)
**ABI**		1.06 (0.15)	1.02 (0.18)	1.09 (0.23)	1.06 (0.16)
**HDL**		50.86 (16.55)	40.69 (10.38)	44.95 (14.14)	49.61 (16.33)
**LDL**		129.19 (45.84)	135.15 (42.08)	125.17 (55.72)	129.42 (46.37)
**Triglycerides**		139.58 (99.57)	214.15 (173.76)	226.16 (463.81)	151.52 (162.43)
**Fasting Blood Glucose**		99.63 (16.97)	119.66 (26.82)	238.33 (80.12)	110.39 (49.73)
**FCRS**		1.25 (1.01)	4.86 (1.36)	2.59 (1.76)	1.64 (1.69)

[a] Abbreviations: SE = Standard error; SD = Standard deviation; CESD = Center for Epidemiologic Studies Depression scale; BMI = Body mass index; ABI = Ankle-brachial index; HDL = High-density lipoproteins; LDL = Low-density lipoproteins; FCRS = Framingham Cardiovascular Risk Score

© 2020 Stickel et al. *JAMA Otolaryngology–Head Neck Surgery.*

eTable 12. Estimated odds ratios and 95% confidence intervals of the association between three group cardiovascular risk classification and hearing impairment.[a]

		Hearing Loss >25 dB
		**OR [95%CI]**
**Cardiovascular Risk Groups**		
	Typical	ref
	High CVDR	1.38 [1.02,1.87]
	Hyperglycemia	1.39 [1.03,1.88]
**Age**		1.10 [1.09,1.11]
**Sex**		
	Male	2.12 [1.73,2.59]
**Education**		
	<12	ref
	12	0.73 [0.58,0.91]
	>12	0.59 [0.47,0.73]
**Center**		
	Bronx	ref
	Chicago	0.96 [0.75,1.22]
	Miami	1.04 [0.83,1.30]
	San Diego	0.99 [0.77,1.27]

[a] Abbreviations: OR = Odds ratio; CI = Confidence Interval; CVDR = Cardiovascular risk; dB = decibels

© 2020 Stickel et al. *JAMA Otolaryngology–Head Neck Surgery.*

eTable 13. Associations between hearing impairment, three group cardiovascular risk classification and cognitive function.[a, b]

		M1	M2	M3	M4
		**B [95% CI]**	**B [95% CI]**	**B [95% CI]**	**B [95% CI]**
**B-SEVLT-Sum**					
	**Typical**	ref	n/a	ref	ref
	**High CVDR**	-0.16 [-0.24;-0.07]	n/a	-0.15 [-0.23;-0.06]	-0.12 [-0.20;-0.04]
	**Hyperglycemia**	-0.06 [-0.15;0.02]	n/a	-0.05 [-0.14;0.03]	-0.02 [-0.11;0.06]
	**≤25 dB (Normal)**	n/a	ref	ref	ref
	**>25 dB (Mild or more)**	n/a	-0.15 [-0.22;-0.09]	-0.14 [-0.21;-0.08]	-0.12 [-0.18;-0.05]
	**Intercept**	1.05 [0.87;1.22]	1.04 [0.86;1.22]	0.97 [0.79;1.15]	1.05 [0.87;1.23]
**B-SEVLT-Recall**					
	**Typical**	ref	n/a	ref	ref
	**High CVDR**	-0.14 [-0.22;-0.05]	n/a	-0.13 [-0.22;-0.04]	-0.10 [-0.19;-0.02]
	**Hyperglycemia**	0.01 [-0.06;0.09]	n/a	0.02 [-0.06;0.10]	0.05 [-0.03;0.13]
	**≤25 dB (Normal)**	n/a	ref	ref	ref
	**>25 dB (Mild or more)**	n/a	-0.10 [-0.16;-0.04]	-0.10 [-0.16;-0.03]	-0.07 [-0.13;-0.00]
	**Intercept**	0.98 [0.81;1.16]	1.01 [0.84;1.19]	0.94 [0.77;1.12]	0.96 [0.78;1.15]
**Word Fluency**					
	**Typical**	ref	n/a	ref	ref
	**High CVDR**	-0.13 [-0.23;-0.04]	n/a	-0.13 [-0.23;-0.03]	-0.11 [-0.20;-0.01]
	**Hyperglycemia**	-0.07 [-0.16;0.02]	n/a	-0.06 [-0.15;0.03]	-0.04 [-0.12;0.05]
	**≤25 dB (Normal)**	n/a	ref	ref	ref
	**>25 dB (Mild or more)**	n/a	-0.13 [-0.20;-0.07]	-0.13 [-0.19;-0.06]	-0.10 [-0.17;-0.04]
	**Intercept**	-0.21 [-0.42;0.01]	-0.22 [-0.43;-0.01]	-0.29 [-0.50;-0.07]	-0.38 [-0.60;-0.17]
**Digit Symbol Substitution**					
	**Typical**	ref	n/a	ref	ref
	**High CVDR**	-0.07 [-0.15;0.00]	n/a	-0.07 [-0.15;0.00]	-0.04 [-0.11;0.03]
	**Hyperglycemia**	-0.07 [-0.15;0.00]	n/a	-0.07 [-0.14;0.01]	-0.04 [-0.11;0.04]

© 2020 Stickel et al. *JAMA Otolaryngology–Head Neck Surgery.*

	**≤25 dB (Normal)**	n/a	ref	ref	ref
	**>25 dB (Mild or more)**	n/a	-0.06 [-0.12;-0.00]	-0.06 [-0.11;0.00]	-0.04 [-0.09;0.02]
	**Intercept**	1.26 [1.08;1.45]	1.25 [1.08;1.43]	1.22 [1.04;1.41]	1.00 [0.83;1.17]
**Global Cognition**					
	**Typical**	ref	n/a	ref	ref
	**High CVDR**	-0.13 [-0.19;-0.06]	n/a	-0.12 [-0.19;-0.06]	-0.09 [-0.16;-0.03]
	**Hyperglycemia**	-0.05 [-0.11;0.02]	n/a	-0.04 [-0.10;0.03]	-0.01 [-0.07;0.05]
	**≤25 dB (Normal)**	n/a	ref	ref	ref
	**>25 dB (Mild or more)**	n/a	-0.11 [-0.16;-0.07]	-0.11 [-0.15;-0.06]	-0.08 [-0.13;-0.04]
	**Intercept**	0.78 [0.65;0.92]	0.78 [0.65;0.91]	0.72 [0.58;0.86]	0.67 [0.55;0.80]

[a] Abbreviations: B = Coefficient estimate; CI = Confidence Interval; ref = Reference category; n/a = not applicable; B-SEVLT = Brief–Spanish English Verbal Learning Test; dB = decibels; CVDR = Cardiovascular risk

[b] M1, M2, and M3 are age, sex, education, and Field Center adjusted. M4 include additional adjustment for Hispanic/Latino Background, Income, Marital Status and CESD-10.

eTable 14. Tests for significance of the modifications in the effects of hearing impairment by three group cardiovascular risk classification.[a]

Cognitive Test	F	df
B-SEVLT-Sum	4.539	2
B-SEVLT-Recall	3.537	2
Word Fluency	0.609	2
Digit Symbol Substitution	0.374	2
Global Cognition	2.175	2

[a] Abbreviations: B-SEVLT = Brief–Spanish English Verbal Learning Test; df = Degree of freedom

© 2020 Stickel et al. *JAMA Otolaryngology–Head Neck Surgery.*

eFigure 1. Risk profiles of latent cardiovascular risk classes (3-class solution) derived from the latent profile analyses.

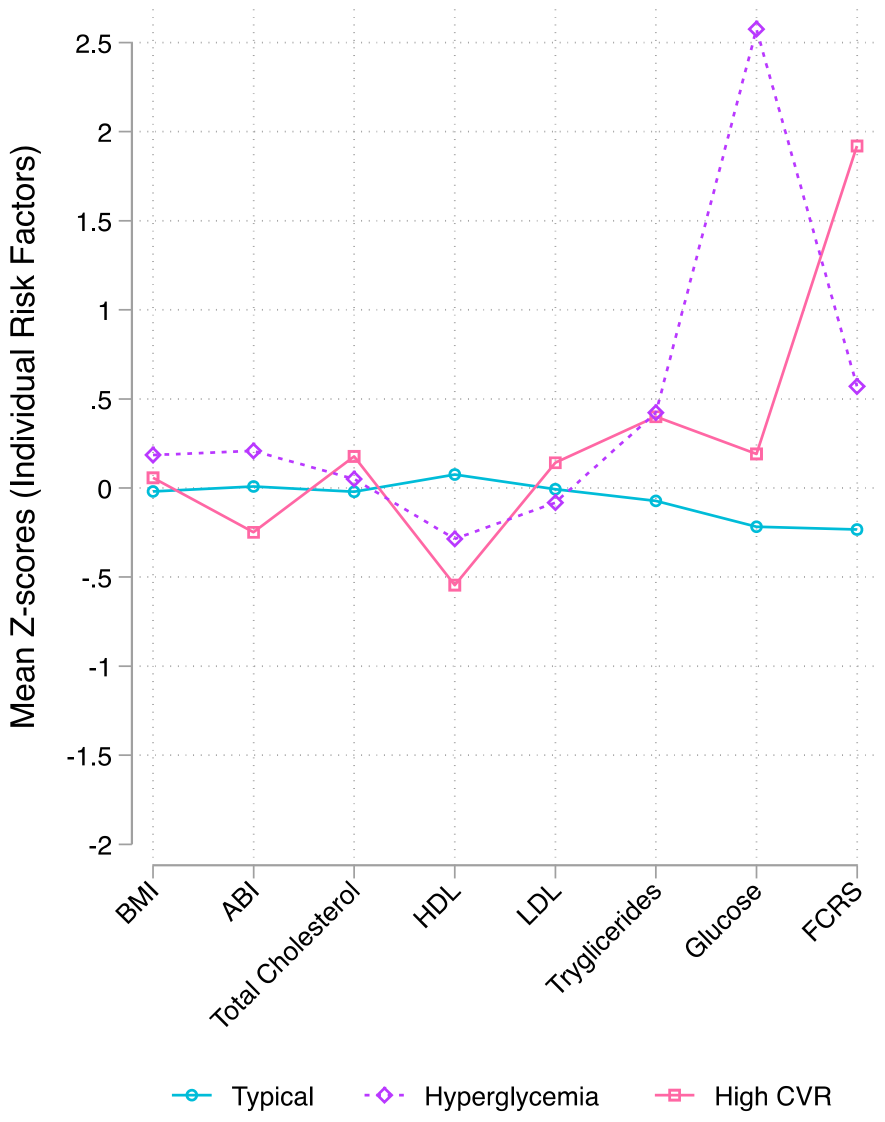

eFigure 1. Legend:
Abbreviations: BMI = Body mass index; ABI = Ankle-brachial index; HDL = High-density lipoproteins; LDL = Low-density lipoproteins; FCRS = Framingham Cardiovascular Risk Score; CVR = Cardiovascular Risk

© 2020 Stickel et al. *JAMA Otolaryngology–Head Neck Surgery*.

eFigure 2. Cognitive performance (z-score) by hearing impairment status.

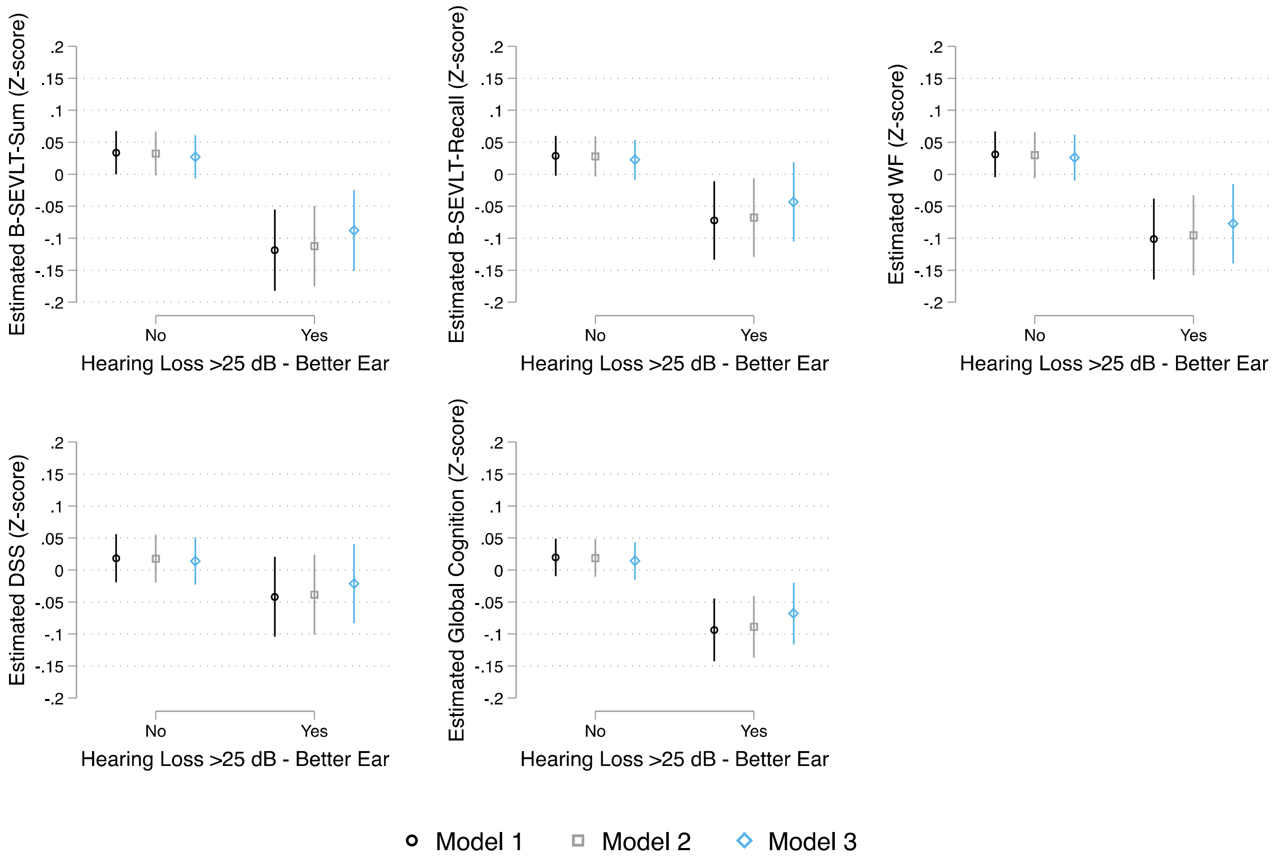

eFigure 2. Legend:
Abbreviations: B-SEVLT = Brief-Spanish English Verbal Learning Test; WF = Word Fluency; DSS = Digit Symbol Substitution.
Model1 adjusted for age, sex, education, and Field Center adjusted. Model 2 includes additional adjustment for cardiovascular risk classification (3-class solution). Model 3 includes additional adjustment for Hispanic/Latino background, income, marital status, and depressive symptoms.

© 2020 Stickel et al. *JAMA Otolaryngology–Head Neck Surgery*.

eFigure 3. Modification in cognitive performance (z-score) by hearing impairment status and cardiovascular risk classification (3-class solution).

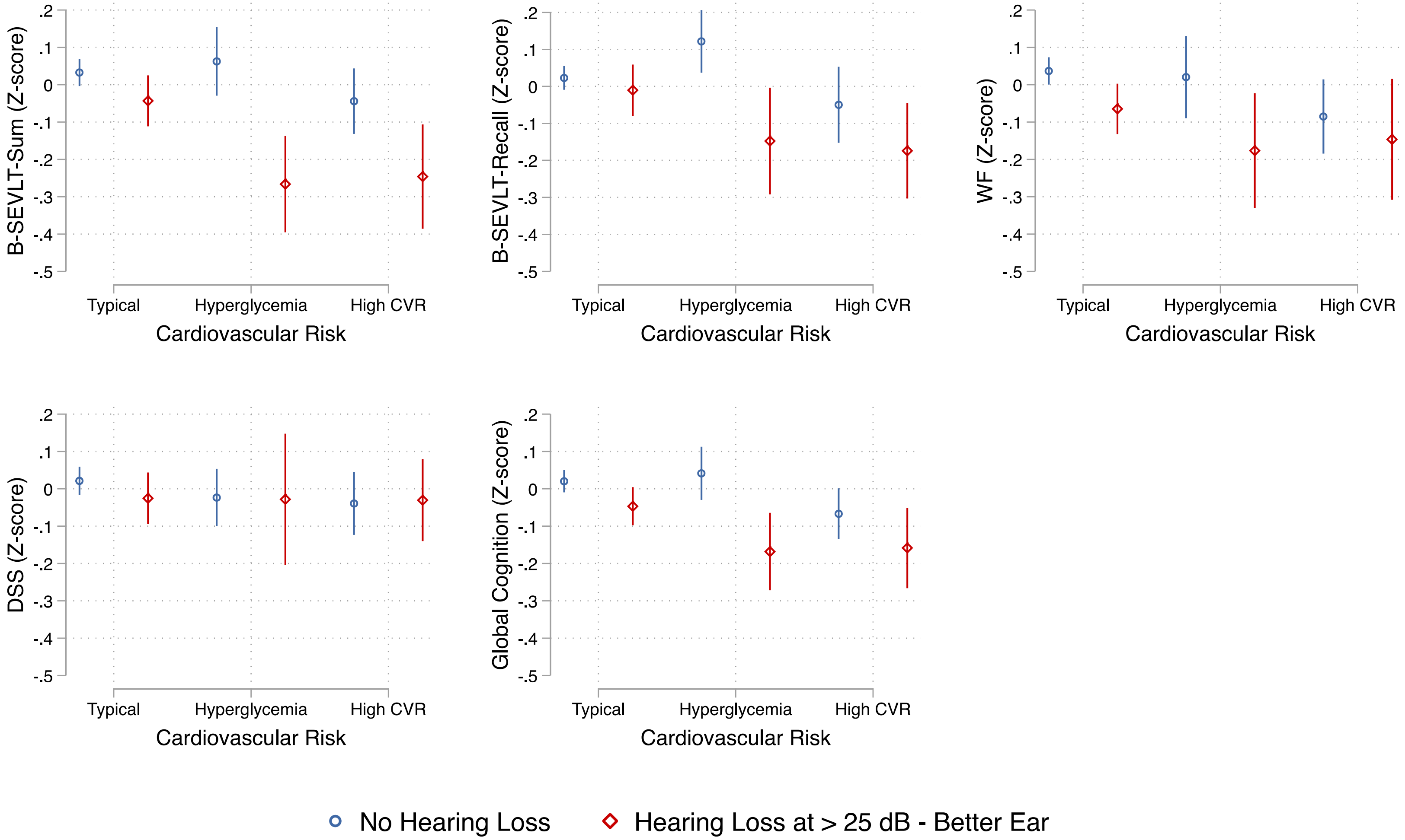

eFigure 3. Legend:
Abbreviations: B-SEVLT = Brief-Spanish English Verbal Learning Test; WF = Word Fluency; DSS = Digit Symbol Substitution.
Models includes adjustment for age, sex, education, Field Center, Hispanic/Latino background, income, marital status, and depressive symptoms.

© 2020 Stickel et al. *JAMA Otolaryngology–Head Neck Surgery*.

REFERENCES

1. Ferguson SL, G. Moore EW, Hull DM. Finding latent groups in observed data: A primer on latent profile analysis in Mplus for applied researchers. *International Journal of Behavioral Development.* 2019;44(5):458-468.
2. Oberski D. Mixture Models: Latent Profile and Latent Class Analysis. In: J. R, M. K, eds. *Modern Statistical Methods for HCI*. Springer, Cham.; 2016:275-287.
3. Masyn KE. Latent class analysis and finite mixture modeling. In: Little T, ed. *The Oxford handbook of quantitative methods* New York, NY: Oxford University Press; 2013:551-611

© 2020 Stickel et al. *JAMA Otolaryngology–Head Neck Surgery.*
